# Cronkhite-Canada Syndrome: an unusual finding of gastro-intestinal adenomatous polyps in a syndrome characterized by hamartomatous polyps

**DOI:** 10.1093/gastro/gou041

**Published:** 2014-06-30

**Authors:** Christopher M. Flannery, John A. Lunn

**Affiliations:** ^1^Department of Gastroenterology, Loma Linda University Medical Center, Loma Linda, CA, USA and; ^2^Department of Gastroenterology, Veterans Affairs Loma Linda Healthcare System Loma Linda, CA, USA

**Keywords:** Cronkhite-Canada syndrome, gastro-intestinal adenomatous polyps, hamartomatous polyps

## Abstract

Cronkhite-Canada syndrome is a rare, hamartomatous polyposis syndrome of unknown etiology. Hamartomatous gastro-intestinal polyps, alopecia, onychodystrophy, cutaneous hyperpigmentation, abdominal pain, diarrhea, and complications of weight loss are typical of the syndrome. In this report, we describe a pathological finding of colonic adenomatous polyposis as opposed to hamartomatous polyposis. We also describe our treatment, long-term therapeutic plan, and the need for further research.

## INTRODUCTION

In 1955 Cronkhite and Canada described an unusual syndrome of polyposis [1]. This syndrome is a rare, non-familial disorder of unknown etiology, associated with hair loss, nail changes, cutaneous hyperpigmentation, hamartomatous gastro-intestinal polyps, diarrhea, weight loss, abdominal pain, and complications of malnutrition [2, 3].

Since first reported by Cronkhite and Canada, there have been about 450 additional cases described in the literature, in which the incidence of adenomatous changes is reported as rising from 40%, based on 2008 data, to 71% as most recently reported in 2012 [4, 5]. Cronkhite-Canada syndrome (CCS) has a low reported cancer potential of only 9–15% [5–8]. Serrated adenoma-associated malignant neoplasm has been reported in some Japanese cases, and a single case in Korea [9]. Despite different medical and surgical therapies, no optimal treatment method is yet known. Nutritional support, antibiotics, corticosteroids, anabolic steroids, histamine-receptor antagonists and surgical treatment have all been used with varying degrees of success. Unfortunately, controlled therapeutic trials have not been possible because of the rarity of the syndrome [5, 6, 10, 11].

In this report we describe a case of CCS with extensive adenomatous change (found in every polyp removed) within innumerable hamartomatous colonic polyps.

## CASE PRESENTATION

A 70-year-old Caucasian male with a history of chronic obstructive pulmonary disease (COPD), depression, hypothyroidism, chronic kidney disease, congestive heart failure, and atrial fibrillation presented to the emergency department with fever, bilateral flank pain, and myalgia. He also reported benign polyps, found on colonoscopy six years ago and, after further questioning, a *clostridium difficile* infection three years previously. He was subsequently admitted for sepsis and found to a have *Klebsiella* urinary tract infection and bacteremia. His hospital course was complicated by acute chronic systolic congestive heart failure, and respiratory distress from healthcare-associated pneumonia. Incidental findings while hospitalized included iron deficiency anemia, vitamin A deficiency, vitamin B deficiency, and vitamin D deficiency.

**Figure 1.**
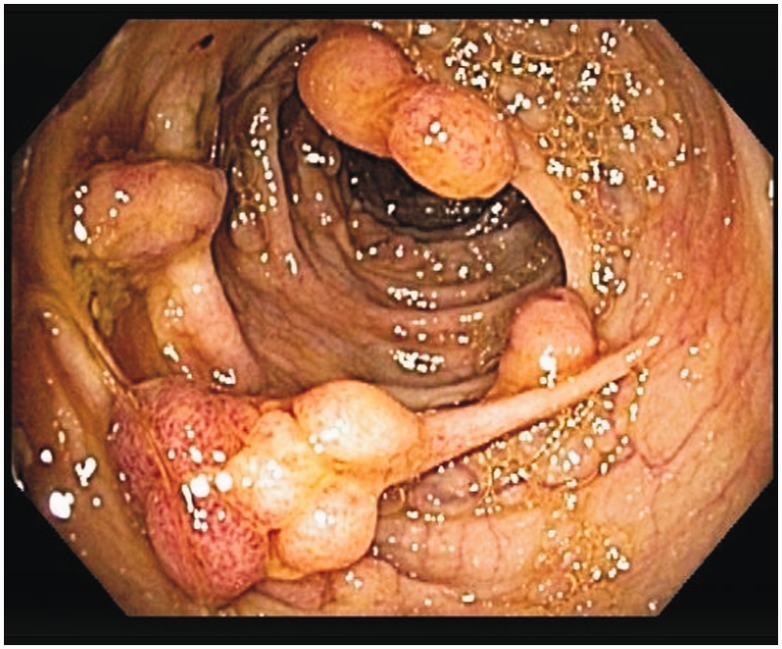
Innumerable, large, frond like, pedunculated polyps throughout the colon.

**Figure 2.**
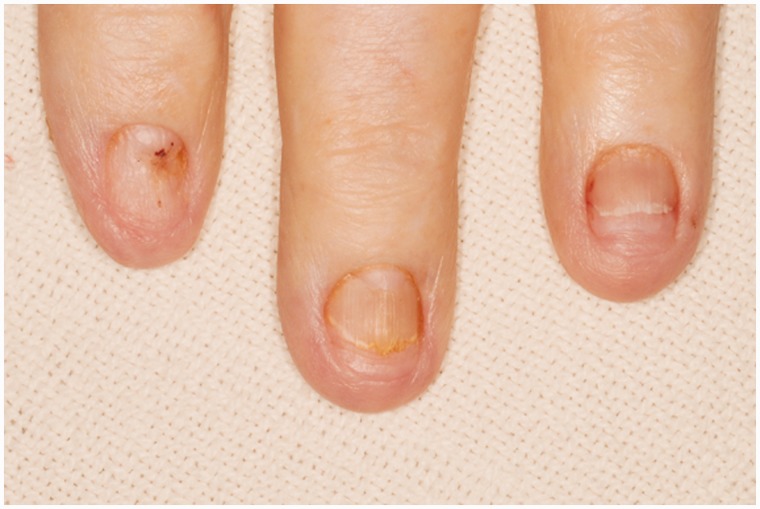
Onychodsytrophy.

**Figure 3.**
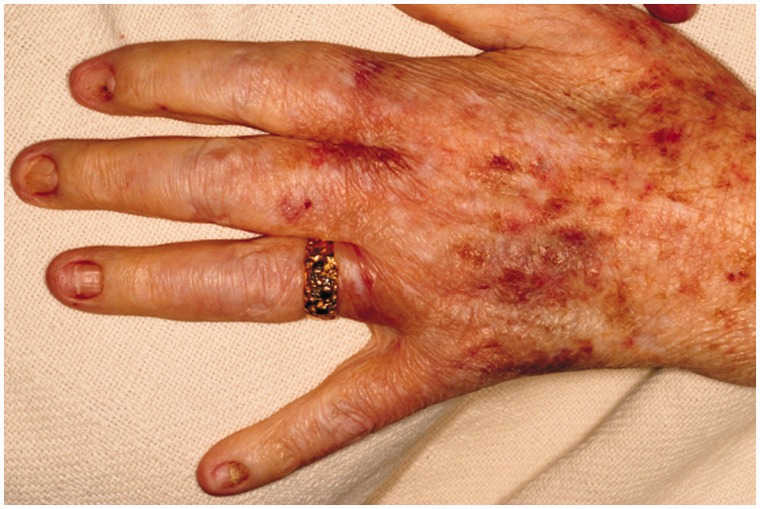
Cutaneous hyperpigmentation.

**Figure 4.**
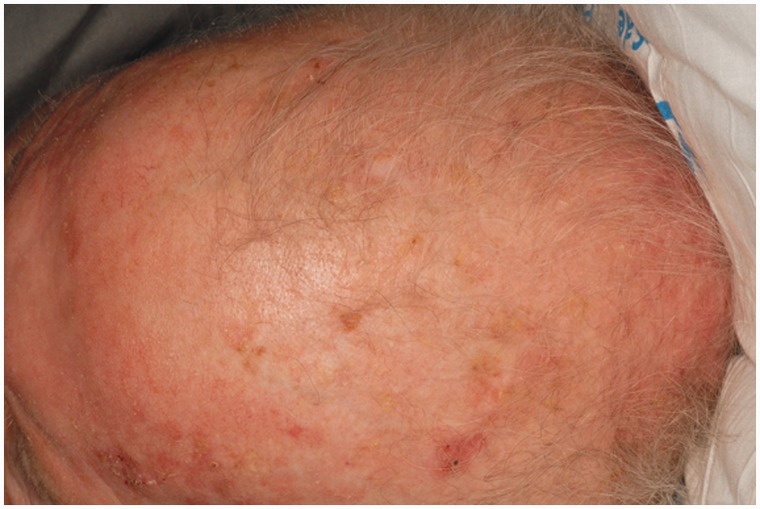
Alopecia.

**Figure 5.**
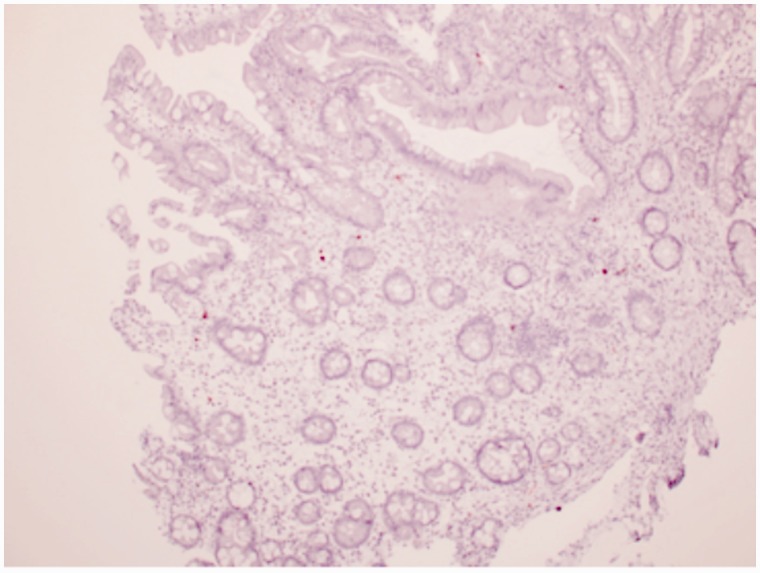
Polyp with hamartomatous characteristics at the base.

**Figure 6.**
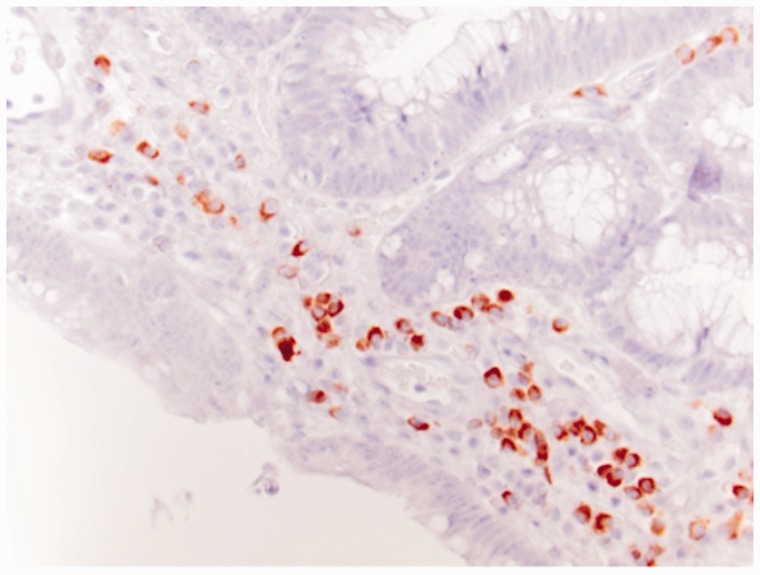
Immunohistochemical stains for IgG4 showed scattered plasma cells with positive staining reaction.

Prior to this presentation, he had recently enrolled with a new primary medical doctor who, ordered laboratory tests that included a fecal immunochemical test (FIT). The patient was FIT-positive, so an outpatient colonoscopy was ordered for cancer screening. While still hospitalized, but after significant improvement, his previously-scheduled colonoscopy was performed. Findings of innumerable, large, frond-like, pedunculated polyps were found throughout the colon (Figure 1). The appearance was of an undefined polyposis syndrome. During the colonoscopy it was observed that the patient had onychodsytrophy (Figure 2), cutaneous hyperpigmentation (Figure 3), and alopecia (Figure 4). Multiple hot-snare polypectomies and random biopsies were taken from the cecum, transverse colon, splenic flexure, and rectum. Microscopic findings of the specimens demonstrated adenomatous changes in a background milieu of classic CCS. The polyps confirmed hamartomatous characteristics only at the base (Figure 5). Immunohistochemical stains for IgG4 showed scattered plasma cells with positive staining reaction (Figure 6).

**Figure 7.**
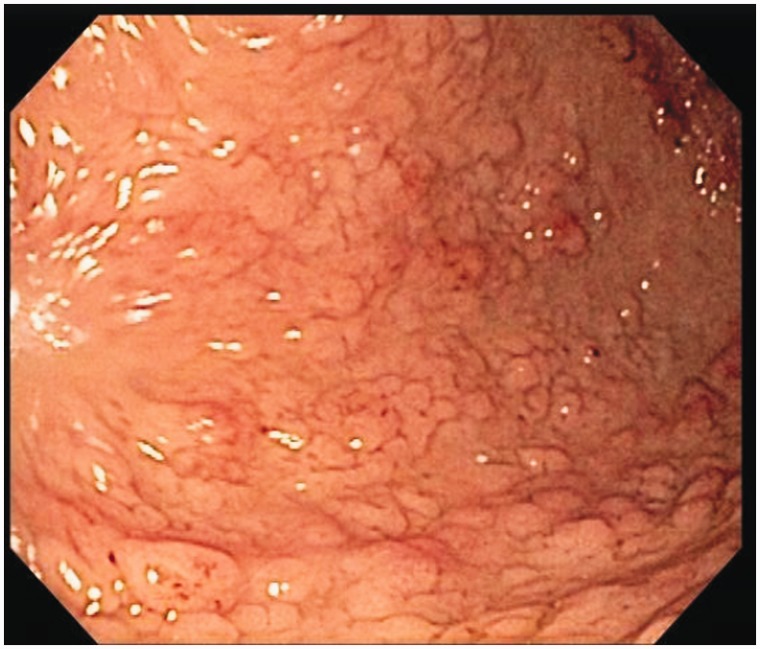
Nodular mucosa was found in the body of the stomach, antrum, and prepyloric region.

**Figure 8.**
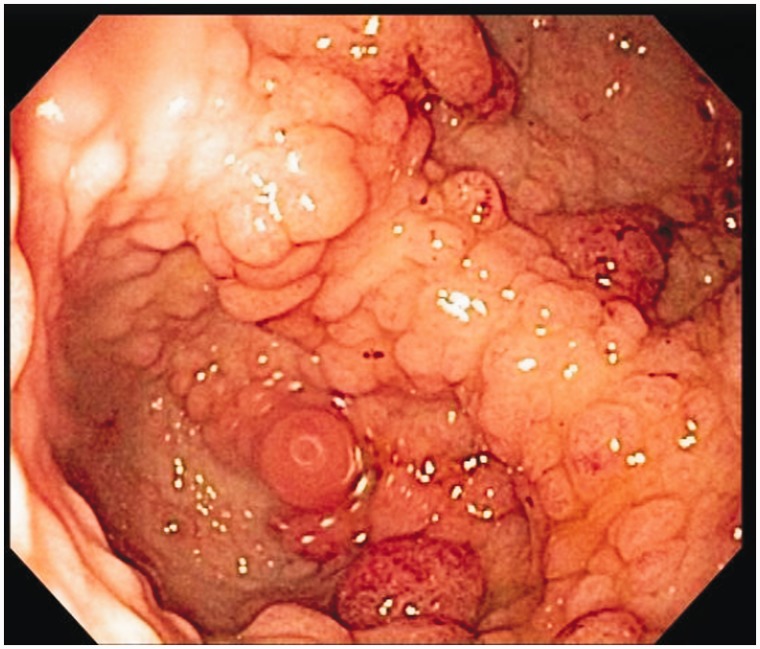
The antrum had a more polypoid appearance.

Several days later, an esophago-gastroduodenoscopy was performed, further demonstrating evidence of the disease. Nodular mucosa was found in the body of the stomach, stomach antrum, and prepyloric region (Figure 7). The body of the stomach showed nodularity, which became more prominent distally. The antrum had a more polypoid appearance (Figure 8). Biopsies were taken and microscopic findings revealed benign mucosa consistent with foveolar expansion (hamartomas) of the gastric body, antrum and pylorus, as described in previous CCS cases.

The patient was initially treated with optimizing nutrition and corticosteroids but developed suicidal inclinations. Azathioprine was chosen to replace the corticosteroids and thoughts of self-harm ceased.

## DISCUSSION

Patients with CCS typically present with diarrhea, weight lost, and complaints of integumentary abnormalities. In this case our patient was initially scheduled for colonoscopy because of a positive fecal immunochemical test; however, prior to his scheduled appointment he was admitted to the hospital for sepsis related to a *Klebsiella* urinary tract infection and *Klebsiella* bacteremia, probably *sequelae* of malnourishment and dehydration.

Gastro-intestinal polyps are found in 52–96% of patients with CCS and range in location from the stomach to the rectum [2]. These polyps are classically described as Hamartomas; however, the incidence of adenomatous change as seen in our patient has been reported to be as high as 71% (10 out of 17 patients), as reported by Sweetster [5]. Other polyp features can include myxoid expansion of the *lamina propria*, and increased eosinophils [2, 12]. In some patients, immunostains of the polyps are IgG4-positive, as in this one [5]. The estimated colon cancer risk is 9–15%, and the five-year mortality rate is as high as 55%, with most deaths being due to gastro-intestinal bleeding, sepsis, and congestive heart failure [5–8].

Alhough this is a non-familial disorder of unknown etiology, hypotheses suggest an immune-mediated disorder. Tests on CCS patients have shown poor results from lymphocyte mitogen stimulation and immunostains positive for IgG4 [5, 13, 14].

To date, there is no effective, specific treatment. As this is a rare disease, no controlled therapeutic trial results are available in the literature; however, treatment options have included optimizing nutrition, glucocorticoids, azathioprine, anabolic steroids, antibiotics, surgery, and even eradication of *H. pylori* plus colectomy [10]. Despite the different attempts at treatment, the most effective therapy appears to be the optimizing nutrition. In addition to treating our patient’s sepsis with hydration and antibiotics, we initiated immunosuppressive therapy and consulted a registered dietitian to optimize the patient's nutrition regimen. After initiating treatment with prednisone the patient became suicidal; subsequently this was discontinued and his suicidal ideation abated. Azathioprine treatment was then begun, as the patient deferred any surgical intervention. Our long-term treatment options—in addition to nutrition support and azathioprine—will be esophago-gastroduodenoscopy and colonoscopy every six months, with resection of polyps greater than 1 cm, also assessing response to treatment and early detection of neoplasia.

In summary, there is much to be learnt with regard to this rare syndrome. Diagnosis, variants of the syndrome, and optimal treatment are yet to be known. An unusual feature of this case is that 100% of polyp specimens from the colonoscopy demonstrated adenomatous changes. We report this case to raise awareness of adenomatous polyps in CCS. Continued evidence suggests this polypoid disease is not purely harmatomatous, with the largest series to date reporting an incidence of adenoma as high as 71% [9]. We recommend aggressive colorectal screening and long-term nutritional optimization.

**Conflict of interest:** none declared.

## References

[1] Cronkhite LW, Canada WJ (1955). Generalized gastro-intestinal polyposis: an unusual syndrome of polyposis, pigmentation, alopecia, and onychotrophia. N Engl J Med.

[2] Daniel ES, Ludwig SL, Lewin KJ (1982). The Cronkhite-Canada syndrome: an analysis of the pathologic features and therapy in 55 patients. Medicine (Baltimore).

[3] Anderson RD, Patel R, Hamilton JK (2006). Cronkhite-Canada syndrome presenting as eosinophilic gastroenteritis. Proc (Bayl Univ Med Cent).

[4] Calva D, Howe JR (2008). Hamartomatous polyposis syndromes. Surg Clin North Am.

[5] Sweetser S, Ahlquist DA, Osborn NK (2012). Clinicopathologic features and treatment outcomes in Cronkhite-Canada syndrome: support for autoimmunity. Dig Dis Sci.

[6] Ward EM, Wolfsen HC (2003). Pharmacological management of Cronkhite-Canada syndrome. Expert Opin Pharmacother.

[7] Chadalavada R, Brown DK, Walker AN (2003). Cronkhite-Canada syndrome: sustained remission after corticosteroid treatment. Am J Gastroenterol.

[8] Yashiro M, Kobayashi H, Kubo N (2004). Cronkhite-Canada syndrome containing colon cancer and serrated adenoma lesions. Digestion.

[9] Yun SH, Cho JW, Kim JW (2013). Cronkhite-Canada syndrome associated with serrated adenoma and malignant polyp: a case report and a literature review of 13 Cronkhite-Canada syndrome cases in Korea. Clin Endosc.

[10] Kato K, Ishii Y, Mazaki T (2013). Spontaneous regression of polyposis following abdominal colectomy and helicobacter pylori eradication for Cronkhite-Canada syndrome. Case Rep Gastroenterol.

[11] Sampson JE, Harmon ML, Cushman M (2007). Corticosteroid-responsive Cronkhite-Canada syndrome complicated by thrombosis. Dig Dis Sci.

[12] Zbuk KM, Eng C (2007). Hamartomatous polyposis syndromes. Nat Clin Pract Gastroenterol Hepatol.

[13] Goto A (1995). Cronkhite-Canada syndrome: epidemiological study of 110 cases reported in Japan. Nihon Geka Hokan.

[14] Lin HJ, Tsai YT, Lee SD (1987). The Cronkhite-Canada syndrome with focus on immunity and infection. Report of a case. J Clin Gastroenterol.

